# FLAIR vascular hyperintensity is associated with functional outcome in patients with ischemic stroke receiving endovascular treatment: a meta-analysis

**DOI:** 10.3389/fneur.2024.1497504

**Published:** 2024-11-08

**Authors:** Chunyan Wang, Chuanliu Wang, Yongjun Ni

**Affiliations:** ^1^Department of Radiology, The Quzhou Affiliated Hospital of Wenzhou Medical University, (Quzhou People’s Hospital), Quzhou, China; ^2^Department of Neurology, The Quzhou Affiliated Hospital of Wenzhou Medical University, (Quzhou People’s Hospital), Quzhou, China; ^3^Department of Radiology, Jiaxing Maternity and Child Health Care Hospital, Jiaxing, China

**Keywords:** vascular hyperintensity, FLAIR, ischemic stroke, endovascular thrombectomy, meta-analysis

## Abstract

**Background:**

Fluid-attenuated inversion recovery (FLAIR) vascular hyperintensity (FVH) might be useful for predicting and functional outcome in ischemic stroke patients after endovascular thrombectomy (EVT), but its clinical benefit remains controversial. Thus, this study aimed to evaluate the association of FVH on prognosis in ischemic stroke patients who received EVT.

**Methods:**

PubMed, Embase, Cochrane Library, Web of Science, and Wanfang databases were searched for potentially eligible studies published up to March 2024. Pooled standard mean difference (SMD), risk ratios (RR) with 95% confidence intervals (CI) were employed to assess the association of FVH on prognosis in ischemic stroke patients who received EVT. All statistical analyses were conducted using STATA 12.0 software.

**Results:**

A total of 10 studies were included in our study. The results indicated that higher FVH score were associated with better prognosis (SMD: 0.80, 95% CI 0.63–0.97). Moreover, the presence of FVH was significant associated with better functional outcome in ischemic stroke patients who received EVT (RR: 0.68, 95% CI, 0.58–0.79).

**Conclusion:**

The current meta-analysis suggests that FVH is related the prognosis of ischemic stroke patients after EVT.

## Introduction

Stroke is the second leading cause of death and disability globally, with ischemic stroke accounting for 60–80% of all strokes, playing a heavy burden on society because of the associated high mortality and disability rates (1). Endovascular thrombectomy (EVT) is becoming a frontline treatment for patients with ischemic stroke (2). Identifying irreversible infarcted areas and salvageable penumbral zones is crucial for guiding treatment decisions. Previous studies have suggested that the ischemic penumbra only suffers functional impairment rather than structural damage, and timely reperfusion of occluded vessels can prevent the occurrence of cerebral infarction (3). Therefore, the identification and evaluation of diseased vessels and infarcted lesions in patients with acute ischemic stroke are crucial for clinical treatment and prognosis assessment. In recent years, digital subtraction angiography has been considered the gold standard for evaluating collateral circulation due to its high spatial and temporal resolution (4). However, considering the complications of irreversible kidney damage caused by contrast agent injection, as well as the inability of patients with renal insufficiency or contrast agent allergies to undergo contrast agent imaging, it is necessary to find a less invasive imaging method to evaluate collateral circulation.

Fluid-attenuated inversion recovery (FLAIR) vascular hyperintensity (FVH) is considered a potential surrogate marker for collateral status (5). FVH is characterized by punctate, tubular, or serpentine high signal intensity along the course of vessels on FLAIR sequences (6). The main mechanism of its formation is the slow flow velocity in the artery proximal to severe stenosis or occlusion or in the distal pial collateral artery (retrograde flow) after severe stenosis or occlusion, leading to the disappearance of flow voids within the vessel (7). Many studies have confirmed the association of FVH with the severity of circulatory and hemodynamic impairment (8, 9). However, its clinical significance remains controversial. Some studies have reported that pretreatment FVH is associated with good functional outcomes in ischemic stroke patients undergoing endovascular therapy, while the authors of other studies have reached the opposite conclusion (10–13). Therefore, the aim of this meta-analysis was to determine the relationship between FVH and the prognosis of ischemic stroke patients who received endovascular therapy.

## Methods

The study was performed in accordance with Preferred Reporting Items for Systematic Reviews and Meta-Analyses (PRISMA) (14).

### Search strategy

Published studies were retrieved from PubMed, Embase, Cochrane Library, Web of Science, and Wanfang databases. Databases were searched from inception to March 2024. The search strategy was based on a combination of the following keywords: stroke, cerebral infarction, endovascular thrombectomy, arterial hyperintensity, and hyperintense vessels. There was no language or other search restrictions. The references of related articles were also manually searched to ensure that no studies were omitted.

### Inclusion and exclusion criteria

The inclusion criteria were as follows: (1) patients (aged ≥18) were diagnosed with ischemic stroke and received endovascular therapy; (2) received brain MRI test after admission and before endovascular therapy; (3) prospective or retrospective cohort study. The exclusion criteria were: (1) preclinical studies; (2) duplicated publications; (3) relevant data on FVH not available; (4) reviews, conference abstracts, or case reports.

### Data collection and quality evaluation

Two authors independently reviewed the title, abstracts, and full text of the retrieved studies to determine their eligibility for inclusion. Then, relevant information from the eligible studies was extracted into a pre-specified form. The data extraction included: first author, country, year of publication, study design, age, sample sizes, and site of occlusion. The methodological quality of each study was assessed independently by two researchers using the Newcastle Ottawa scale (NOS) (15).

### Statistical analyses

All statistical analyses were performed using Stata software (v.12.0). The forest plots were generated to present the standard mean difference (SMD), risk ratios (RR), and 95% confidence intervals (CI). The heterogeneity of the studies was evaluated by *I*^2^ statistics (25% = low, 50% = medium, 75% = high). A fixed-effects model was used for heterogeneity <50%, and a random-effects model was used for heterogeneity >50%. Sensitivity analysis was conducted by removing studies one by one to assess the influence of individual studies on the estimate of the overall effect. Publication bias was determined using Begg and Egger’s test for funnel plot asymmetry. A two-sided *p*-value of <0.05 indicates statistical significance.

## Result

### Study selection and characteristics

**Figure 1:**
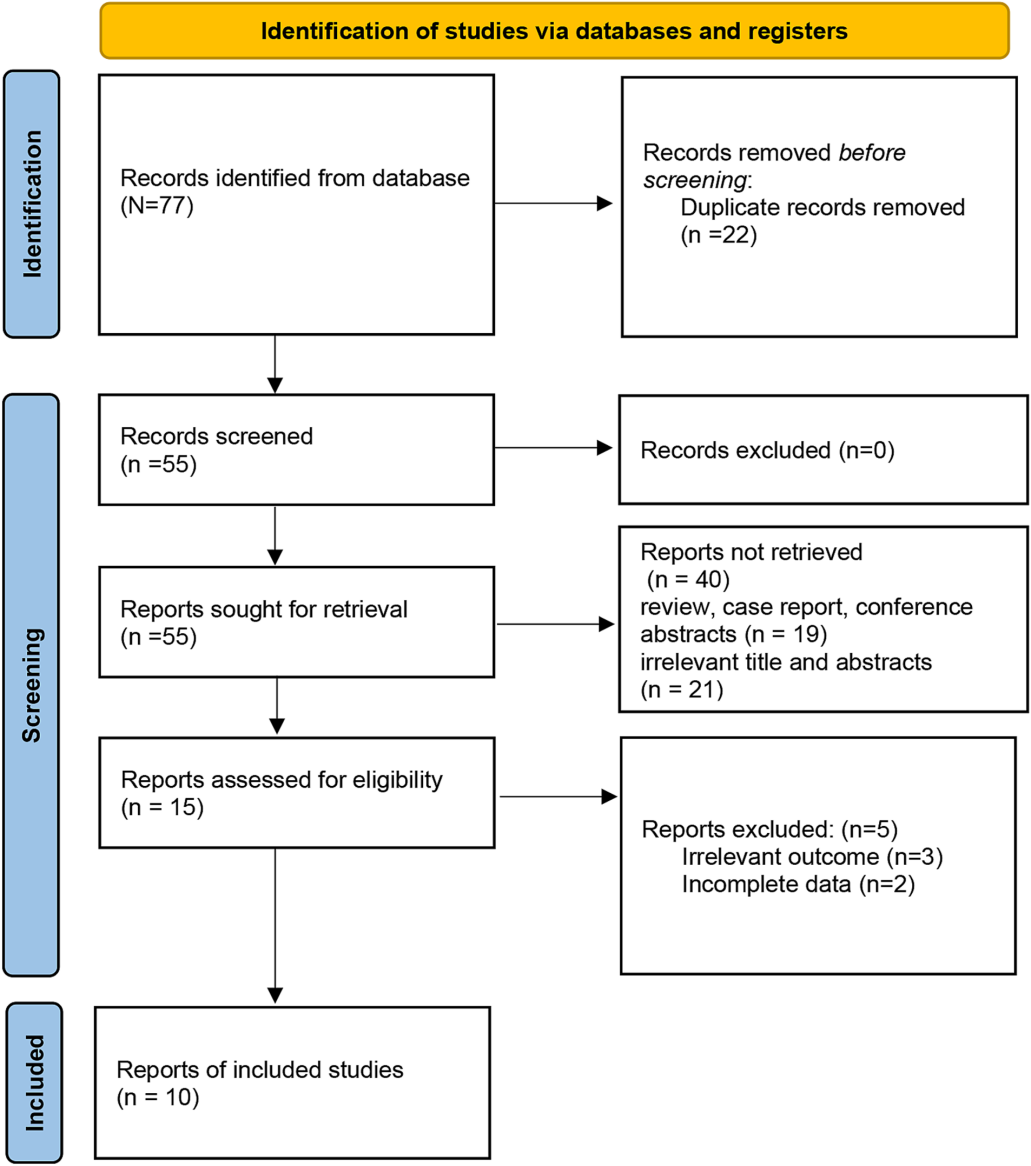
Flow diagram for selection of studies.

Based on the search strategy, the initial literature search identified 77 relevant studies, of which 22 were excluded due to duplication. After title and abstract screening, 15 articles remained. During the full-text assessment, 5 studies were excluded because irrelevant outcome (*n* = 3) and incomplete data (*n* = 2). Finally, 10 eligible articles were selected for this meta-analysis (10–13, 16–21) (Figure 1). The included studies were published between 2016 and 2023, and the sample size ranged from 53 to 316. A total of 5 studies were prospective design, while 5 studies were retrospective. The detailed characteristics of the included articles are summarized in Table 1. In addition, the NOS scores ranged from 6 to 8, suggesting a moderate and high quality of all included studies (Table 2).

**Table 1:** Basic characteristics of the included studies.

Author, year	Country	Study design	Gender (M/F)	Age (years)	Sample size	Site of occlusion
Derraz, 2021	France	Retrospectively cohort	170/146	68.5	316	M1 MCA, ICA, Tandem
Wang, 2020	China	Prospective cohort	41/31	69.69	72	LVO
Jiang, 2020	China	Prospective cohort	29/25	70.06	53	M1 MCA, ICA
Jiang, 2019	China	Prospective cohort	36/23	67.74	59	M1 MCA, ICA
Li, 2019	China	Retrospectively cohort	23/14	69.41	37	LVO
Xu, 2020	China	Retrospectively cohort	41/22	70.74	63	LVO
Liu, 2016	USA	Prospective cohort	29/72	66.25	101	M1 MCA
Nave, 2018	Germany	Prospective cohort	89/27	73	116	M1 MCA
Legrand, 2019	France	Retrospectively cohort	54/46	60.5	100	M1 MCA
Kaewumporn, 2023	Thailand	Retrospectively cohort	50/70	66.93	110	LVO

ICA, internal carotid artery; LVO, large vessel occlusion; MCA, middle cerebral artery.

**Table 2:** Newcastle Ottawa scale.

First author	Is the case definition adequate?	Representativeness of the cases	Selection of controls	Definition of controls	Control for important factors	Exposure assessment	Same method of ascertainment for cases and controls	Non-response rate	NOS score
Derraz	1	0	0	1	1	1	1	1	6
Wang	1	0	0	1	2	1	1	1	7
Jiang	1	1	0	1	1	1	1	1	7
Jiang	1	0	0	1	1	1	1	1	6
Li	1	0	0	1	1	1	1	1	6
Xu	1	0	0	1	1	1	1	1	6
Liu	1	1	0	1	1	1	1	1	7
Nave	1	1	0	1	2	1	1	1	8
Legrand	1	0	0	1	1	1	1	1	6
Kaewumporn	1	1	0	1	1	1	1	1	7

### Association of FVH with functional outcome

**Figure 2:**
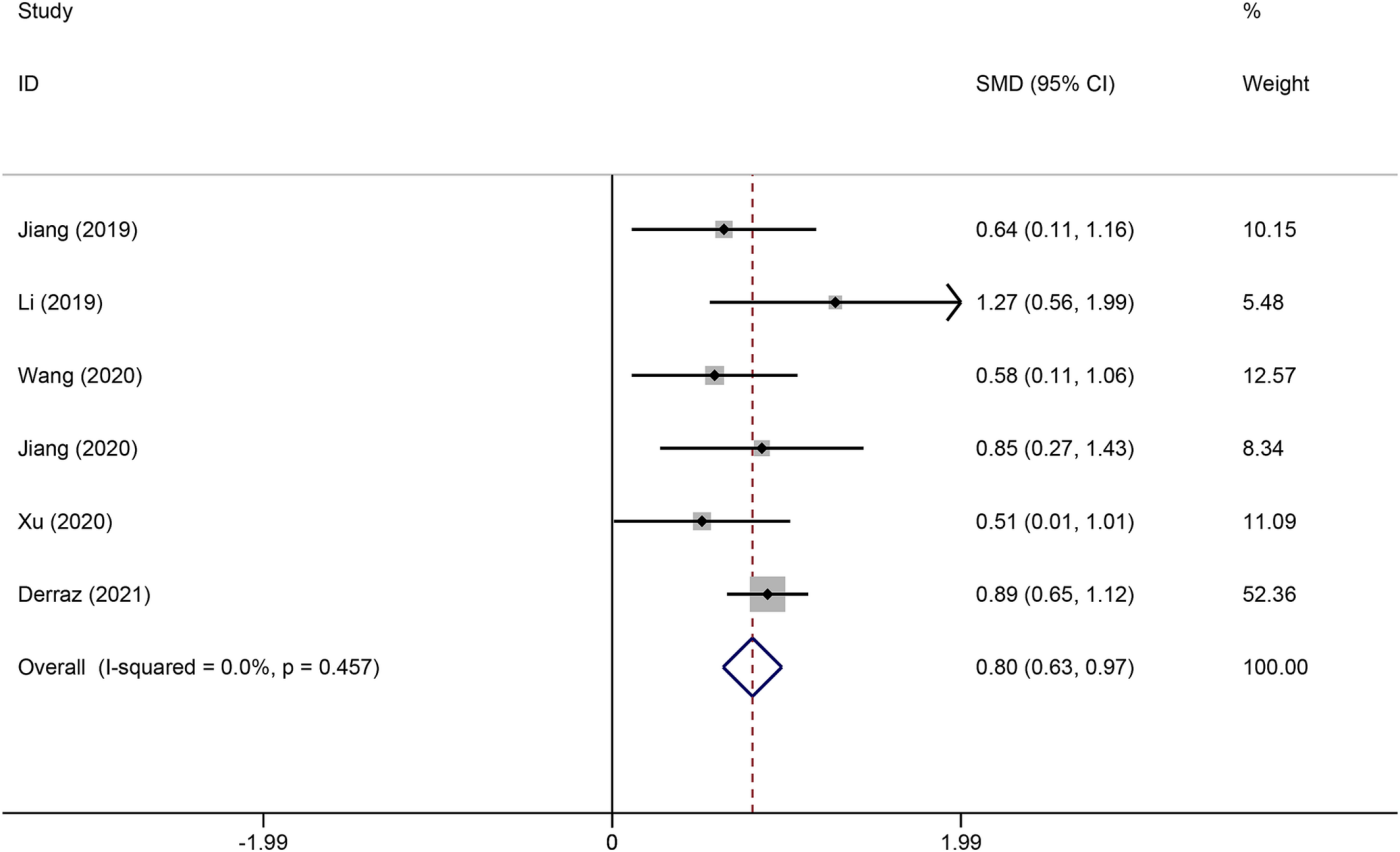
Meta-analysis of associations between FVH score and outcome.

**Figure 3:**
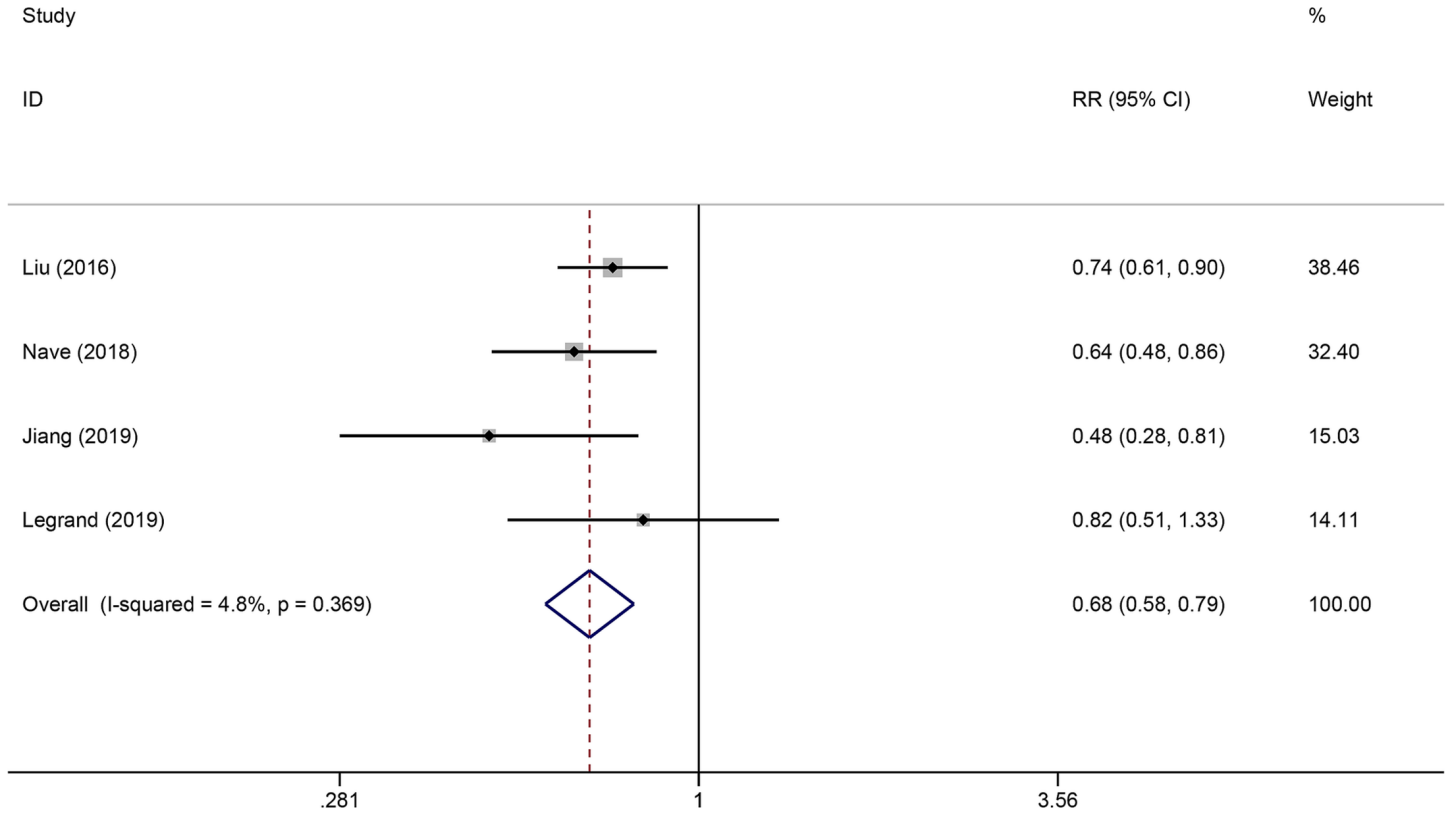
Meta-analysis of associations between FVH presence and outcome.

Six studies reported the FVH score in the good functional outcome and poor functional outcome groups. As shown in Figure 2, higher FVH scores were associated with better prognosis (SMD: 0.80, 95% CI 0.63–0.97, *p* < 0.001). No significant heterogeneity across studies was found (*I*^2^ = 12.6%, *p* = 0.334). Four studies reported the relationship between FVH presence and unfavorable functional outcomes. Overall, compared with no FVH, the presence of FVH was significantly associated with better functional outcomes who had endovascular therapy (RR: 0.68, 95% CI, 0.58–0.79, *p* < 0.001) (Figure 3).

### Sensitivity analysis and publication bias

**Figure 4:**
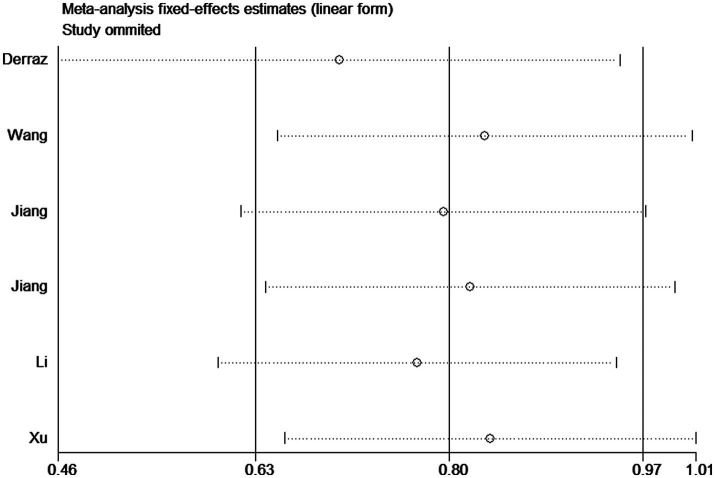
Sensitivity analysis for associations between FVH score and outcome.

Sensitivity analyses were conducted using the study-by-study approach, and no significant changes were found, indicating that results were reliable (Figure 4). In addition, Begg and Egger tests did not show a significant publication bias (Begg test, *p* = 0.348; Egger test, *p* = 0.703).

## Discussion

The FLAIR sequence has high sensitivity and specificity for evaluating brain parenchymal lesions and subarachnoid spaces, and has been widely used in the diagnosis of ischemic Stroke and intracranial stenosis (22). The advantages of the FLAIR sequence, such as its noninvasiveness, low time consumption, and few contraindications, are inherent to FVH. FVH can provide information on arterial stenosis, tissues at risk of infarction, and collateral circulation (23). The limitation of FVH is its unclear clinical significance, which is the focus of this study. FVH can reflect the retrograde filling status of the pial collateral circulation and has been confirmed by cerebral angiography (24). It has great reliability, practicality, and reproducibility and can be widely used in clinical practice and trials. Current meta-analyses suggest that baseline FVH is associated with the prognosis of ischemic Stroke patients undergoing endovascular treatment based on 10 relevant studies involving 961 patients.

FVH has previously been classified based on its appearance (dot sign, linear high signal, serpentine high signal), location (proximal and distal FVH), and score (low and high FVH) (7, 25). As mentioned earlier, the FVH score reflects the number of pial collateral vessels recruited during acute arterial flow interruption in the brain. Therefore, the higher the FVH score is, the more collateral vessels are shown on FLAIR MR images, indicating more highly detectable signals (26). On the other hand, the degree of FVH is a scale that reflects the degree of collateral vessel recruitment (i.e., quality). Therefore, the subtle appearance of high signals on MRI is considered to reflect a lower efficiency of collateral vessel recruitment, whereas prominent FVH may represent more severe collateral vessel involvement (27). The extent of the collateral response may be driven by the quality of cerebral blood flow, which is related to the degree of vascular response and the overall burden of cerebrovascular microangiopathy (including white matter lesions) (28). It should be noted that some variables may affect the FVH. Previous studies have found that MRI parameters such as echo time (TE) and flip angle (FA), as well as periodically rotated overlapping parallel lines with enhanced reconstruction techniques are independent factors that affect FVH intensity. Additionally, with the increase of TE, the signal intensity of FVH decreases (29). Since 3D-FLAIR is less sensitive to slow flow than 2D-FLAIR, the signal of FVH may be mask obscured in acute stroke patients, which is possibly due to the reduction of refocusing FA (30). In addition, contrast agents can also hinder the detection of FVH, such as gadolinium significantly reduced FVH (31).

Within 44 h of stroke onset, the prevalence of FVH ranges from 1.1 to 24%, increasing to 75.9–100% when severe stenosis or occlusion of the MCA or ICA is present (32). Derraz et al. (27) suggested that FVH scores are associated with improved outcomes and may be helpful for patients choosing reperfusion therapy. However, the observation of persistent FVH after reperfusion therapy may indicate ongoing vascular occlusion and hemodynamic compromise (7). Therefore, patients with FVH may be more susceptible to hemodynamic instability than those without FVH. This difference may be related to adverse outcomes in FVH patients during this period. Some studies that re-examined MR imaging in the hyperacute phase support this hypothesis (5, 7). A previous study revealed that the persistent presence of FVH on follow-up MR images was associated with decreased blood flow signal intensity on MRA, while the absence of FVH was visible when the blood flow signal intensity on MR images recovered (33). However, the observation of decreased FVH after reperfusion therapy indicates successful reperfusion, smaller infarct volumes, and a better prognosis. Nave et al. (20) suggested that a low FVH was associated with a better 90-day functional prognosis. However, Li et al. (19) found no correlation between the FVH score and 90-day clinical functional outcomes, indicating that the FVH score cannot be used as a predictor of short-term clinical outcomes. Our meta-analysis results revealed that FVH scores can be used to predict the prognosis of ischemic stroke patients undergoing endovascular treatment, with patients with high FVH scores having a better prognosis.

Good collateral vessels are associated with increased odds of a larger volume of surviving brain tissue by maintaining the ischemic penumbra and are thus associated with better clinical outcomes (11). Patients with FVH-DWI mismatch had smaller initial DWI volumes upon admission, lower DWI volume growth at follow-up, and smaller final DWI volumes (18). This finding is consistent with that of Lee et al. (34), who reported that patients with more prominent distal high-signal vessels had smaller initial and 24-h ischemic lesion volumes. In contrast, Hohenhaus et al. (35) reported that patients with an FVH score of >4 had larger initial DWI lesions and final infarct volumes. The differences between studies may be due to the use of different FVH scoring methods. Currently, there is no standardized classification for FVH, with three main approaches commonly used to evaluate FVH. For example, Lee et al. (34) divided FVH in the proximal middle cerebral artery (MCA) into two categories based on the presence or absence of FVH. For the distal MCA territory, FVH is classified into three grades: none, subtle, and prominent. Olindo et al. (36) used the origin of the M1 segment of the MCA as a reference, fluid-attenuated inversion recovery (FLAIR) images are divided into 10 layers. If no FVH is present in any layer, the score is 0; the presence of FVH in one layer is scored as 1, continuing similarly, with a maximum total score of 10. The Alberta Stroke Program Early Computerized Tomography Score (ASPECTS) divided the MCA territory into 7 regions on FLAIR imaging. Each region showing FVH is assigned 1 point (37). Recent research has found that differences in FVH classification may be responsible for these inconsistent results (34). Our meta-analysis revealed that the presence of FVH was associated with favorable recovery in patients who received endovascular therapy.

Several limitations in this study should be mentioned. First, relatively few studies included outcomes other than FVH scores, and more research is needed to further confirm the conclusions. Second, in the present study, more retrospective studies with many confounding factors and a small overall sample size were included, which may have led to a greater risk of bias. Third, there were differences in stroke subtypes and etiology among the included studies, all of which could impact the prognosis of the patients. Due to the limited number of studies analyzed, further analysis could not be conducted using these inconsistent data.

In summary, the current meta-analysis suggested that FVH is closely related to the prognosis of ischemic stroke patients after EVT. We believe that FVH may be a useful prognostic biomarker, although more well-designed and larger sample sizes and high-quality studies are needed to further validate our findings.

## Data Availability

The original contributions presented in the study are included in the article/supplementary material, further inquiries can be directed to the corresponding author.
